# Supplementary material for: The sodium-proton exchangers sNHE and NHE1 control plasma membrane hyperpolarization in mouse sperm
Source: bioRxiv. 2024 Mar 6:2024.03.04.583310. Preprint. [Version 1] doi: 10.1101/2024.03.04.583310 (PMC10942401; doi:10.1101/2024.03.04.583310)
Supplement: Supplement 1 [file media-1.pdf]

SUPPLEMENTARY INFORMATION

Supplementary Figure 1.

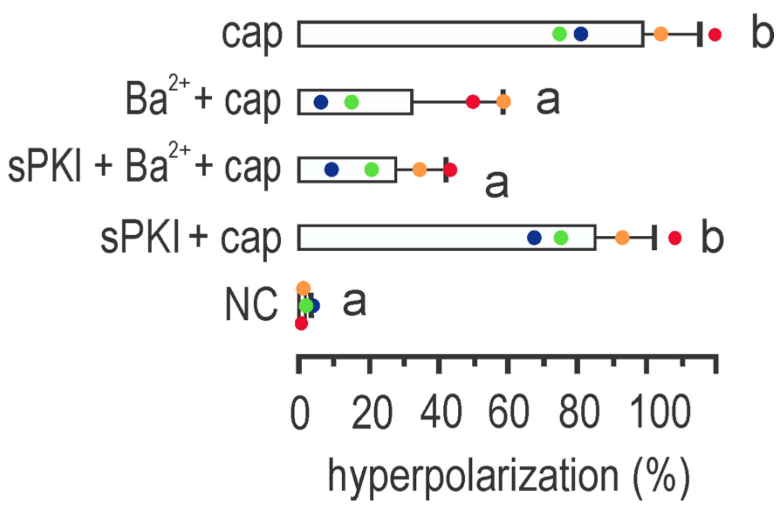

Legend

Supplementary Figure 1. Ba^{2+} blocks *E*m hyperpolarization even in the presence of sPKI. Sperm E_m measurements obtained after incubation for 60 min in capacitating conditions containing either 1 mM $BaCl_2$ or 15 µM sPKI or a combination of both. Results are expressed as a normalization of percentage of hyperpolarization considering mean NC and cap values as 0% and 100%, respectively (mean ± SEM; n=4); different letters indicate statistically significant differences (p<0.05).
